# Reply to: “Enhancing Life-course Analyses of Financial Disadvantage and Depressive Mood Through Longitudinal Design and Causal Inference”

**DOI:** 10.2188/jea.JE20250625

**Published:** 2026-05-05

**Authors:** Hiroshi Murayama, Erika Kobayashi, Hidehiro Sugisawa, Benjamin A. Shaw, Jersey Liang

**Affiliations:** 1 Research Team for Social Participation and Healthy Aging, Tokyo Metropolitan Institute for Geriatrics and Gerontology, Tokyo, Japan; 2 Graduate School of International Studies, J. F. Oberlin University, Tokyo, Japan; 3 Division of Community Health Sciences, University of Illinois, Chicago, Chicago, IL USA; 4 University of Michigan School of Public Health, Ann Arbor, MI USA

To the Editor,

We sincerely thank Dr Quan Zhang for his thoughtful comments on our study,^1^ presented in his recent letter.^2^ His suggestions on longitudinal modeling, addressing unmeasured confounders, and assessing policy implications have provided valuable directions for advancing life-course epidemiological research. We have responded to each point below.

**1. Strengthening life-course analyses using longitudinal data**

We agree that the cross-sectional nature of our study, which relied on retrospective socioeconomic information, limits causal inference. However, In Japan, few longitudinal surveys have comprehensively traced socioeconomic status (SES) from childhood to late life. Among available data, the National Survey of the Japanese Elderly (NSJE), which we analyzed in our study, has followed multiple cohorts since 1987 and serves as a foundation for future analyses of SES trajectories. Nonetheless, because it targets individuals aged 60 years and older, early life circumstances cannot be directly captured. The NSJE may also serve as a valuable platform for validating retrospective measures of life-course SES in older adults.

In life-course epidemiology, retrospective reports are considered useful complementary measures, and several studies support their validity. Wadsworth and Kuh showed that recalled childhood SES corresponds well with archival records,^3^ and Batty et al found substantial agreement between adult recall and recorded social class.^4^ These findings indicate that retrospective data can complement prospective measures when they are unavailable.

Dr Zhang’s proposed weighting framework appears primarily conceptual, assuming a longitudinally observed SES, rather than a retrospective recall. We consider this conceptual approach as complementary to the empirical trajectory modeling based on existing national cohorts. His idea serves as a methodological roadmap for future research that combines repeated SES assessments with trajectory modeling to more rigorously examine temporal sequences.

**2. Accounting for unmeasured confounders and mediators**

As Dr Zhang noted, our study lacked data on prior depression or medication use, which is a limitation we acknowledged. Nevertheless, longitudinal studies have consistently shown robust associations between SES trajectories and mental health, even after extensive adjustments.^5^ Linking survey data with administrative health or prescription records could substantially improve confounding controls. Although such a link remains challenging in Japan, it represents an important and promising direction.

Structural equations or cross-lagged panel models can be used to clarify the bidirectional relationship between SES and depressive mood. Using multiple NSJE waves, it would be possible to examine the mediating pathway linking SES change to depressive mood through health behaviors, advancing from statistical associations to causal inference. In this sense, we view Dr Zhang’s recommendations as a methodological guide for future longitudinal studies, rather than analytic strategies directly applicable to our current dataset.

**3. Evaluating policy and economic implications**

Dr Zhang’s proposal to apply a Markov model to policy simulations is conceptually innovative. However, from a public health perspective, an empirical step is required to determine whether the health effects of social mobility can be reproduced through real-world interventions. In Japan, lifetime financial trajectories are shaped by education, employment stability, reemployment support, social security, and pension systems.

Recent evidence shows that income-support and employment-promotion policies improve mental health.^6^ When assessing cost-effectiveness, broader societal benefits, such as functional independence, frailty prevention, and healthcare-cost reduction, should be included within a social-return-on-investment framework. Our study provides a foundation for such analyses; thus, the next step is to incorporate modifiable social factors (including education, social participation, and continued employment) into causal models.

In summary, we thank Dr Zhang for highlighting important methodological and translational directions that extend beyond the aims of our cross-sectional analysis in life-course social epidemiology. Our study demonstrates, in the Japanese context, that upward socioeconomic mobility is linked to lower depressive mood later in life. Future research should combine longitudinal evidence, causal inference approaches, and policy modeling to verify this relationship and establish a scientific basis for social policies that disrupt cumulative disadvantages and promote mental well-being in aging societies.
